# Fournier’s Gangrene Surgical Reconstruction: A Systematic Review

**DOI:** 10.3390/jcm13144085

**Published:** 2024-07-12

**Authors:** Pietro Susini, Gianluca Marcaccini, Jessica Efica, Maria Teresa Giuffrè, Ruggero Mazzotta, Corso Caneschi, Roberto Cuomo, Giuseppe Nisi, Luca Grimaldi

**Affiliations:** 1 Plastic Surgery Unit, Department of Medicine, Surgery and Neuroscience, University of Siena, 53100 Siena, Italy; g.marcaccini@student.unisi.it (G.M.); j.efica@student.unisi.it (J.E.); m.giuffre2@student.unisi.it (M.T.G.); roberto.cuomo@unisi.it (R.C.); giuseppe.nisi@unisi.it (G.N.); luca.grimaldi@unisi.it (L.G.); 2 Division of General Cardiology, Careggi University Hospital, 50134 Florence, Italy; ruggero.mazzotta@unifi.it; 3 Unit of Urological Robotic Surgery and Renal Transplantation, Careggi University Hospital, 50134 Florence, Italy; corso.caneschi@unifi.it

**Keywords:** Fournier gangrene, Fournier gangrene reconstruction, Fournier gangrene treatment, Fournier gangrene plastic surgery, necrotizing fasciitis, necrotizing fasciitis and reconstruction

## Abstract

Fournier’s gangrene (FG) is a rare form of necrotizing fasciitis of the perineal, genital, or perianal region. It is characterized by an aggressive course and high mortality rate, over 20%. FG demands immediate treatment including resuscitation maneuvers, intravenous antibiotic therapy and early surgical debridement. **Background/Objectives**: The gold-standard treatment for FG is surgical reconstruction. However, up to date, no precise guidelines exist. Thus, we decided to systematically review the literature, focusing on FG contemporary approaches to reconstructive surgery, aiming to analyze the various reconstructive strategies and their specific indications. **Methods**: A systematic review was carried out according to the PRISMA statement by searching various databases from April 2014 to April 2024, using the terms ‘‘Fournier Gangrene OR Fournier Gangrene Reconstruction OR Fournier Gangrene Treatment OR Fournier Gangrene Plastic Surgery OR Necrotizing Fasciitis OR Necrotizing Fasciitis AND Reconstruction”. The eligibility criteria included original studies aimed at discussing FG reconstruction with at least three clinical cases. **Results**: The final synthesis included 38 articles, and 576 reconstructions were described. Of these, 77.6% were minimally invasive strategies (direct closure, secondary healing, grafts, and local random flaps), while more invasive reconstructions (loco-regional flaps based on known vascular anatomy) were adopted in 22.4%. No free flaps were reported. **Conclusions**: FG requires immediate medical interventions including broad-spectrum antibiotic therapy, surgical debridement, adjuvant therapies, and reconstructive surgeries. Taking into account the anatomical characteristics of the inguinal-crural region, skin grafts and local random flaps could offer versatile and effective reconstructions for most FG cases, while the more invasive strategies should be reserved for very few cases. Future research is warranted to define an FG dedicated reconstruction protocol.

## 1. Introduction

Fournier’s gangrene (FG), first documented by Jean-Alfred Fournier in 1883 [1], is a rare form of necrotizing fasciitis of the perineal, genital, or perianal region [2]. FG commonly affects males above 50 years old. Less frequently, at a ratio of 10:1, it occurs in children and females [3]. The latter experience a significantly higher mortality rate due to the different anatomy and greater susceptibility to peritonitis and retroperitonitis [4]. Indeed, female sex is a recognized independent negative prognostic factor, together with cardiovascular disease, diabetes, and overall patient frailty [5,6,7].

FG is characterized by an aggressive course and a high mortality rate of over 20% [8]. It requires immediate intervention, including resuscitation maneuvers, intravenous antibiotic therapy, and early surgical debridement [8]. Various reconstructive surgeries can be considered, aiming to cover the testicles, preserve testicular function, and restore acceptable cosmetic results, while minimizing morbidity and mortality. Surgery is the gold standard for FG treatment. However, the appropriate timing and surgical strategy are still debated, and no precise guidelines exist [9,10].

In the present review, we electively focus on FG contemporary approaches to reconstructive surgery, aiming to analyze various reconstructive strategies and their specific indications. Brief reports on etiopathogenetic factors, medical treatments, and regional anatomy are also mentioned.

## 2. Materials and Methods

### 2.1. The Data Sources and Search Strategy

Following the PRISMA statement for Systematic Reviews [11], the recent literature was searched on the PubMed (MEDLINE), EMBASE, Cochrane, Web of Science, and Scopus databases from April 2014 to April 2024 using the terms ‘‘(Fournier Gangrene) OR (Fournier Gangrene Reconstruction) OR (Fournier Gangrene Treatment) OR (Fournier Gangrene Plastic Surgery) OR (Necrotizing Fasciitis) OR (Necrotizing Fasciitis AND Reconstruction)”. An extensive list of terms to describe the target population based on the PICO acronym was formulated:

P (population)—Fournier’s Gangrene syndrome;

I (intervention)—surgery and surgical reconstruction;

C (comparator)—control group, non-surgical strategy, and medical treatment;

O (outcomes)—reconstruction outcomes and complications assessment. 

This systematic review was registered in the International Prospective Register of Systematic Reviews (PROSPERO), ID: CRD42024555882.

### 2.2. Study Selection

The inclusion criteria were original studies (observational studies or randomized controlled trials) discussing the surgical treatment of FG. Case series were included if they reported a minimum of three FG cases. Studies were excluded if they were animal studies, review articles or meta-analyses, books and documents, case reports, letters to the editor, and papers not written in English. Inconclusive or descriptive evidence was not included. Following title and abstract screening, we established whether publications met the selection criteria. Furthermore, when title and abstract screening alone was unclear, the full text was reviewed and compared to the selection criteria. The bibliographical references were also screened. The included articles were then subjected to a full-text review and tested with the selection criteria. After study selection, data extraction, and critical appraisal, the collected data were brought to the attention of the senior author (LG) for final approval and any disagreement resolution. Accordingly, the selected papers were re-examined and finally included to present the information in this review.

## 3. Results

**Figure 1:**
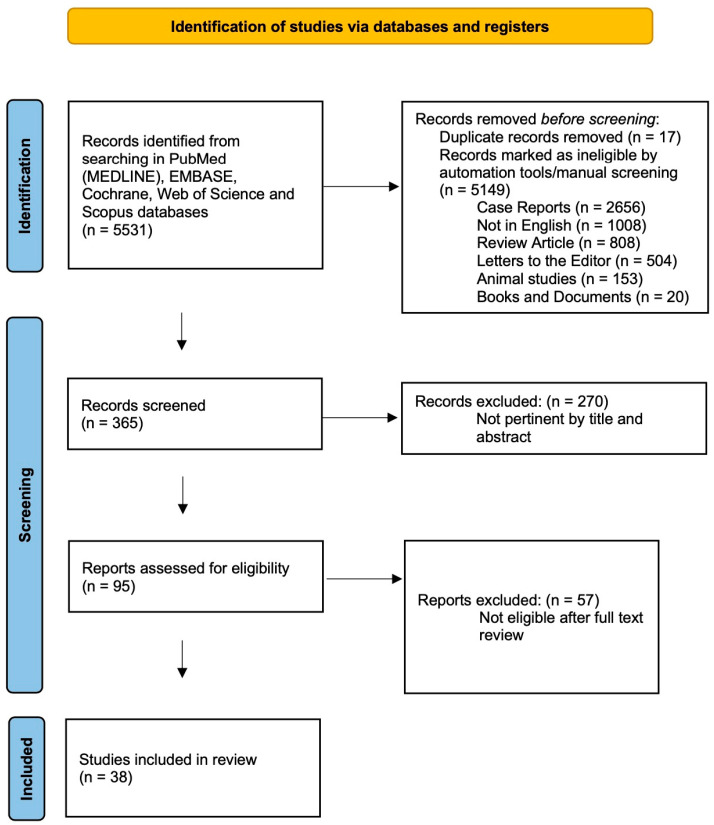
PRISMA 2020 flow diagram summarizing research results.

Based on the established keywords, the primary research revealed a total of 5531 articles (Figure 1). These were compared with selection criteria. By using PubMed’s automatic search tools and manual screening, 2656 case reports, 1008 articles not written in English, 808 reviews and meta-analyses, 504 letters to the editor, 153 animal studies, and 20 books/documents were excluded. Seventeen duplicates were also excluded, and 365 remaining articles were assessed for relevance based on their titles and abstracts; as a result, 95 potentially eligible original articles were selected and fully reviewed. Of these, 59 articles that were not relevant to the aim of this study were excluded. Finally, 38 articles met the selection criteria and were included in this review (Table 1). 

Overall, we identified 719 cases of FG subjected to surgical treatment: 478 males (92.6%) and 37 women (7.4%). Data on comorbidities were available for 510/719 patients (71.4%). Of these, diabetes mellitus was observed in 207/510 (40.5%), cardiovascular disease in 31/510 (6.0%), obesity in 23/510 (4.5%), smoking in 22/510 (4.3%), alcoholism in 21/510 (4.1%), renal failure in 19/510 (3.7%), immunosuppression in 15/510 (2.5%), cirrhosis in 13/510 (2.5%) paraplegia in 6/510 (1.1%), and neoplasm in 6/510 (1.1%). The extension of FG defect was specified for 370/719 (51.5%). Of these, 212 (57.8%) were limited to the penile, scrotal, or vulvar region, 58 (15.7%) were perineal lesions, 100 (27.0%) had extra-perineal extension. 

The reconstructive strategy was available for 593/719 (82.5%). Of these, reconstruction involved direct closure in 35/593 (5.9%), healing by secondary intention in 113/593 (19.1%), skin grafts in 219/593 (37.4%), and loco-regional flaps in 223/593 (37.9%). None of the studies reported the use of free flaps: 0/593 (0%). Loco-regional flaps included random flaps in 90/223 (40.4%) and flaps based on known vascular anatomy in 133/223 (59.6%). 

Overall, a minimally invasive reconstructive strategy (including direct closure, secondary healing, grafts, and local random flaps) was achieved in 460/593 (77.6%), while more invasive strategies represented by loco-regional flaps based on known vascular anatomy or free flaps were used in 133/593 cases (23.4%). 

Data on complications were reported for 272/719 (37.8%). Of these, complications occurred in 21.3%, mostly local complications, such as wound dehiscence, distal flap necrosis, and wound infection (Table 2).

## 4. Discussion

FG is a serious clinical condition that typically occurs in frail patients. The source of infection may be genital, perineal, or genitourinary, but atypical onset has been documented, such as after acute pancreatitis [4,48]. Commonly reported risk factors include male sex, immunosuppression, chronic alcoholism, and prolonged immobilization [5,6]. Moreover, cardiovascular disease, obesity, and diabetes mellitus play a central role [5,6]. Thus, the control and treatment of these are once again crucial in improving a patient’s prognosis. 

In such a scenario, a new class of drugs, the Sodium-glucose Cotransporter-2 (SGLT2) inhibitors, has recently become a topic of debate. First introduced as antidiabetic drugs, SGLT2 inhibitors such as canaglifozin, empaglifozin, and dapaglifozin now represent one major option for the treatment of heart failure. They represent class I recommendation drugs according to the most recent European Society of Cardiology (ESC) guidelines, regardless of the presence or absence of diabetes mellitus [49,50]. Notably, a particular relationship between SGLT2 inhibitors and FG has been reported [51]. Specifically, in 2019, the U.S. Food and Drug Administration (FDA) identified 55 cases of FG in patients treated with SGLT2 inhibitors [52]. Due to the FG onset, serious consequences occurred, and three patients died [52]. 

Since these drugs lower glycemia by preventing urine glucose reabsorption, glycosuria may occur, possibly promoting genital mycotic infections. This latter may trigger FG [53,54,55]. There is still uncertainty on this topic; however, given the relevance of these drugs, future research on SGLT inhibitors and FG should be carried out.

### 4.1. Clinical Presentation and Diagnosis

FG manifests as a wide range of clinical symptoms that initially consist of localized pain, swelling of the affected area, redness, and warmth. As the infection spreads, fever, chills, and general weakness may occur, up to septic shock. Atypical onset such as isolated penile edema with normal skin appearance has also been documented [56]. Therefore, it is crucial to consider FG in various scenarios of swelling, fever, leukocytosis, or disproportionate pain during examination, particularly in elderly patients. Additional signs such as bruising, crepitus, and bullae in the genital region are also reported [57]. 

Due to the urgency of the condition, FG diagnosis primarily relies on clinical judgment, and any suspicion justifies a prompt surgical consultation [58]. Laboratory findings include leukocytosis, increased serum creatinine levels, and metabolic acidosis [59]. Blood cultures are crucial to identify the polymicrobial etiology and guide an effective antibiotic treatment. Imaging methods like CT scans may offer detailed visualization of the perineal structures, allowing for retroperitoneum examination, where FG may extend [60]. Among the various scoring systems used for FG assessment of severity and outcomes, the Fournier Gangrene Severity Index (FGSI) and the Sequential Organ Failure Assessment (SOFA) are the most reliable to predict in-hospital mortality [61]. Continuous research has led to progressive improvements in FG management, with the mortality rate decreasing to 7.3% in the last 20 years [27,62].

### 4.2. Medical Treatment 

Medical treatment plays a central role in FG, and it should be initiated as soon as possible, considering the exponential course of FG and the life-threatening complications. Appropriate initial management includes prompt resuscitation, administration of broad-spectrum antibiotics, and surgical debridement [8]. 

#### 4.2.1. Antibiotic Coverage

FG infection is typically polymicrobial, requiring extended coverage for Staphylococcus, Enterococcus, and E. coli, as well as other Gram-negative pathogens and anaerobes, including Bacteroides and Clostridium species. Occasionally, FG may be caused by atypical pathogens. Thus, antibiotics should be adjusted based on Gram staining and culture results [63].

#### 4.2.2. Early Surgical Debridement 

Early debridement results in improved clinical outcomes, including shorter hospital stays, fewer debridement sessions, and reduced reliance on skin grafting, when compared to conservative approaches of delayed debridement [23,47,64]. Yuki et al. [65] documented the use of ultrasonic debridement as an effective and painless approach for treating chronic wounds containing biofilm, morbid granulation tissue, soft necrotic areas, and, in some cases, hard necrotic tissues resulting from FG. This treatment operates via a cavitation effect: the waves emitted from the device generate numerous microscopic bubbles, promoting emulsification and breakdown of necrotic tissues and biofilms, resulting in effective soft tissue debridement [66]. Despite the limited experience, it could offer considerable advantages.

### 4.3. Adjuvant Therapies 

Appropriate FG management includes adjuvant therapies such as Vacuum-Assisted Closure (VAC) and Hyperbaric Oxygen Therapy (HBOT). The term adjuvant refers to their deferred application to the first debridement surgery. These strategies should be considered in cases of delayed response to conventional therapy or severe infections [8]. In addition, they could offer results both in preparation for and following reconstructive surgery.

#### 4.3.1. Vacuum-Assisted Closure (VAC) 

VAC therapy, also known as Negative-Pressure Wound Therapy (NPWT), involves the application of negative pressure to a wound with a sealed dressing trough a vacuum pump. The technique has been related to several advantages, including increased tissue granulation, wound healing, and limited infections [37,67]. It should represent a core strategy in preparation for FG surgical reconstruction.

#### 4.3.2. Hyperbaric Oxygen Therapy (HBOT)

HBOT involves placing the patient within a pressure vessel or chamber, where they breathe 100% oxygen at atmospheric pressure. HBOT has been used as adjuvant treatment for infectious diseases as it alleviates tissue hypoxia, decreases pathological inflammation, mitigates ischemia–reperfusion injury, and promotes a bactericidal effect [68]. Although its efficacy remains a subject of discussion, studies by Feres et al. [69] and Rizandha et al. [70] indicated a significantly lower mortality rate in FG patients who received adjuvant HBOT compared to those undergoing conventional therapy [70]. In addition, HBOT could have an adjuvant role after reconstructive surgery by improving tissue oxygenation, possibly reducing ischemic complications on surgical flaps and promoting healing [46,70]. Given its promising benefits, HBOT is expected to become increasingly available in hospitals, especially in FG-dedicated medical centers.

### 4.4. Inguinal-Crural Region Anatomy

When considering FG reconstruction, the fasciae and anatomical planes of the inguinal-crural anatomical region play specific roles [71]. The cutaneous and subcutaneous planes including skin, Camper’s superficial fascia, Scarpa’s fascia, and the underlying adipose tissue, accommodate the axial vasculature that nourishes the skin. This latter should be respected to avoid necrosis and failure of any local flaps [71,72]. The muscular, preperitoneal, and peritoneal planes should then be spared to avoid vascular-nervous lesions [71,72]. Notably, surgical dissection on the subcutaneous-supramuscular plane is safe and reliable. It allows for extended recruitment of skin and soft tissues in local flaps, even with random circulation. 

Concerning the scrotum, beneath the skin, there is a thin layer of smooth muscle fibers known as the dartos fascia, followed by three distinct fascial layers derived from the structures of the abdominal wall during embryonic development: the external spermatic fascia (from the external oblique), the cremasteric muscle and fascia (from the internal oblique), and the internal spermatic fascia (from the transversalis fascia) [73,74]. Below these protective coverings resides the tunica vaginalis, directly enveloping each testis [75,76]. 

To reconstruct FG testicular exposure, a reliable and versatile scrotal local advancement flap can be harvested by dissecting between the skin–dartos complex and the underlying external spermatic fascia. This latter should be maintained below, revealing an almost avascular surgical plane.

### 4.5. Reconstructive Surgery 

FG surgical reconstruction varies depending on several factors, such defect size, location, depth, and availability of local tissues [8]. Treatment should be initiated as quickly as possible to cover exposed areas, avoid testicular scarring retraction, and penile deformation [8]. Although extremely complex surgeries are reported in the literature, our analysis reveals that most FG reconstructions are feasible with minimally invasive techniques, while more invasive strategies should be reserved for the most challenging cases. Specifically, out of the 593 highlighted reconstructions, a minimally invasive strategy (including direct closure, secondary healing, grafts, and local random flaps) was achieved in 469/593 (77.6%), while more invasive strategies represented by loco-regional flaps based on known vascular anatomy or free flaps were used in 133/593 cases (23.4%). 

These findings were unexpected. Indeed, FG consequences are particularly severe, up to penile degloving and testicle exposure. In the first instance, these could be misjudged as serious reconstructive challenges due to both extended loss of substance and proximity to the genitourinary apparatuses and related bacterial counts, possibly responsible for infections. Indeed, most surgeons avoid FG reconstruction because of the fear of complications and technical difficulties. However, the inguinocrural anatomical region has a good vascular supply and reliable surgical planes, allowing for satisfactory direct closure, skin grafts, and local random flaps. 

#### 4.5.1. Primary Closure Approach

Primary intention closure involves directly bringing together the edges of the wound when the surrounding tissue is healthy and viable, avoiding extensive tissue removal or manipulation. When dealing with minor FG defects, direct suturing of the remaining scrotal tissue yields the most favorable functional and cosmetic results, and the effectiveness of this procedure is cited in multiple studies [31,33,59]. From our analysis, this strategy was adopted as the sole treatment in 35/593 reconstructions (5.9%). The direct closure approach is relatively easy to perform and should always be considered as a first-line treatment for defects that are limited in size and extension. To improve outcomes, the procedure should be deferred to debridement, considering the increased risk of infections for simultaneous procedures [77]. 

#### 4.5.2. Secondary-Intention Healing

In healing by secondary intention, the wound is left open to granulate and epithelialize without surgical intervention [13,22]. Over 113 cases have been reported in the present literature, accounting for 19.6% of all reconstructions. Specifically, Chalya et al. [45] obtained wound closure by secondary intention in 65 FG patients (77.4%), reporting optimal results with this strategy. It should be considered for loss of substances of limited extent and depth. Despite the limited indications, it allows for minimally invasive and, sometimes, unnecessary interventions.

#### 4.5.3. Skin Grafts

**Figure 2:**
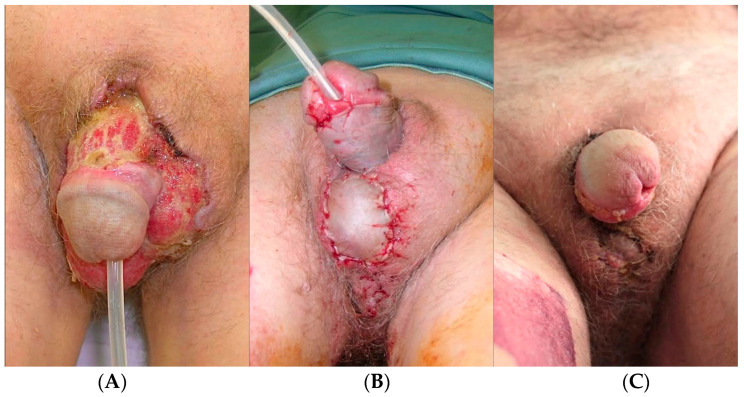
Seventy-two-year-old man with testicular exposure and penile degloving following FG. The patient was first subjected to surgical debridement. After 25 days, the loss of substance was covered with a split-thickness graft. (**A**) preoperative; (**B**) graft inset; (**C**) 1-month follow-up.

Skin grafting involves transplanting healthy skin from a donor area to cover tissue defects. When considering FG, the technique may involve the use of split-thickness or full-thickness grafts. These are commonly employed as deferred procedures, following surgical debridement, when primary intention or secondary healing approaches are not feasible (Figure 2). The inguinal-crural anatomic region has a good vascular supply when compared to other areas, such as the distal leg or the foot [72]. Therefore, grafting represents an excellent and relatively simple strategy. Indeed, in the current literature, it accounts for over 222 procedures and 38% of all reconstructions [12,18,19,22,25,29,38,41,42,43]. Of course, this strategy is inadequate for bone or tedineous exposure, but it is still suitable for most FG cases. 

In addition, porcine and bovine xenografts could be considered [8]. In the field of FG, xenografts are typically regarded as a temporary measure, followed by deferred autografting or reconstruction by flaps [21]. These templates seem promising for direct closure of FG deep perineal, tendinous, and bone lesions, which cannot be reconstructed by direct grafting [8,21]. To date, experience is still limited, but future research should be carried out.

#### 4.5.4. Locoregional Flaps

Local flaps are required in cases of extensive defects, which cannot be managed with primary closure or skin grafting alone. The inguinal-crural region has valuable vascularization, considerable redundancy and good tissue elasticity, ultimately resulting in several reconstructive options [16,39]. When considering FG, over 223 local flaps were described, accounting for 37.6% of all reconstructions. Various local flaps have been described, including both random flaps (90/223—40.4%) and flaps based on known vascular anatomy (133/223—59.6%).

- Random flaps

**Figure 3:**
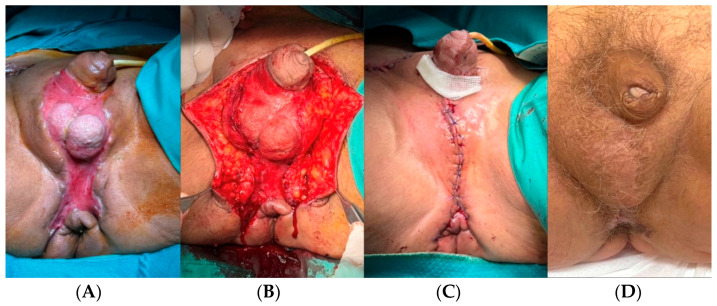
Sixty-five-year-old male patient with testicular exposure following FG. Twenty-one days after surgical debridement, the extensive loss of substance was covered with a bilateral advanced local flap (random vascular anatomy). (**A**) preoperative; (**B**) flap dissection; (**C**) immediate postoperative result; (**D**) one-year follow-up.

Through advancement, rotation, and transposition flaps, it is possible to mobilize tissues with good results [12,13,32,33]. The most commonly reported are local advancement flaps (Figure 3). In FG, these are easy to perform and reliable to cover extended losses of substance, including scrotal reconstructions [12,13,32,33]. 

Moreover, the Limberg flap has been unconventionally described in this anatomical area to provide tension-free transposition closure. Specifically, Dadaci et al. [20] reported 29 FG scrotal reconstructions (including defects in over 50% of the scrotal surface) effectively managed by either double-sided or unilateral Limberg flaps [20]. Despite the limited applications, this unconventional strategy should be considered among the surgeon’s options.

- Locoregional flaps based on known vascular anatomy

The inguinal-crural region has a well-known and constant vascularization that allows for local flaps based on known vascular anatomy [15,35,36]. This represents an effective strategy to manage the major losses of substance. Operations are complex, with long surgeries and serious risks, and must be reserved for well-selected cases. They also require greater surgical training and must be performed in specialized centers. The most commonly used flaps include the anterolateral thigh flap, medial circumflex femoral artery flap, superomedial thigh flap, pudendal thigh flap, groin flap, and McGregor propeller flap.

1 Anterolateral Thigh Flap

The anterolateral thigh flap is a reconstructive option that provides multiple tissue components, including muscle, fascia, skin, or any combination of these [78]. It is effective for covering extensive defects in the lower abdomen, hip regions, groin, perineum, and genital anatomy, including FG consequences [14,15,34,40,44]. It is based on septocutaneous and musculocutaneous perforator branches of the descending branch of the lateral femoral circumflex artery [78]. The pedicle length ranges from 8 to 16 cm, allowing for both pedicled and free-flap reconstructions. Moreover, the flap can be made sensate by including the lateral femoral cutaneous nerve. This latter can be anastomosed to a sensory nerve at the recipient site, possibly restoring tactile and erogenous sensitivity, which might be relevant for these patients [78].

2 Medial Circumflex Femoral Artery Flap

The medial circumflex femoral artery is the first branch of the profunda femoris artery [79]. The vascular pedicle can be included in a local flap, suitable for various reconstructions, including scrotal or femoral triangle reconstructions [80,81]. When attempting FG reconstruction, this flap offers a moderately to large skin paddle while allowing for immediate closure at the donor site. Regrettably, for many individuals, the medial thigh region has a thicker adipose pannus than other parts of the thigh, resulting in bulky reconstructions. Immediate debulking should be avoided, but it may be considered as a deferred procedure. Nevertheless, the medial circumflex femoral artery is also the dominant source vessel to the Gracilis muscle [79,82]. Consistently, a myocutaneous flap can be harvested, suitable for reconstruction of FG deep perineal loss of substances [82].

3 Superomedial thigh flap

The superomedial thigh flap is supplied by three vessels: the superficial branch of the deep external pudendal artery, which constitutes the main blood supply to the flap, the musculocutaneous perforators of the medial circumflex femoral artery, and the branches of the common or superficial femoral artery [17,30]. The flap design should be realized at the upper inner thigh, with the baseline running over and parallel to the tendinous origin of the adductor longus muscle and the upper limit positioned at the origin point of the adductor longus muscle tendon on the pubic tubercle [17,30]. 

The rationale for its use in FG is linked to the anatomical proximity to the defect site. However, given the relatively less constant pedicle, this flap is usually chosen as the second line, when the anterolateral thigh flap or the medial circumflex femoral artery flap is insufficient or unavailable.

4 Pudendal thigh flap

The pudendal thigh flap involves harvesting a fasciocutaneous flap from the upper thigh region to reconstruct damaged or missing tissue of the perineum or genital area [24,83]. It is based on the terminal branches of the superficial perineal artery, from the internal pudendal artery of the internal iliac artery [83]. Specifically, the lateral branches of the perineal artery supply the posteromedial surface of the upper thigh [84], allowing for a local flap that is particularly relevant for FG neo-scrotum reconstructions [24,28]. The main limitations of this technique include the defect site and extension. Indeed, extended and/or FG deep perineal defects may benefit from alternative strategies, such as anterolateral or medial circumflex femoral artery flaps, including myocutaneous variants.

5 Groin flap

The groin flap is vascularized by the superficial circumflex iliac artery [85,86]. The artery originates from the superficial femoral artery 2–3 cm downstream of the inguinal ligament, one-third above the relief of the iliac crest and two-thirds below [85,86]. The flap is designed as an elliptical axial flap, centered on the presumed vessel course. It is rarely used in FG. Indeed, the groin flap has considerable limitations with regard to FG reconstruction, including the variable origin, limited length, and inconsistent caliber of the vascular pedicle. Excessive bulk has also been described in obese patients, possibly impairing penile or vulvar reconstruction. However, it is still valuable for scrotal reconstruction, especially when pudendal thigh flaps are unavailable.

6 McGregor propeller flap

The McGregor propeller flap, first described it in the 1970s by Ian McGregor, is a local flap suitable for extended tissue defects in the inner thigh [87,88]. The flap is designed as a fasciocutaneous propeller flap [88]. In the current literature, only one study reported the McGregor propeller flap for FG genital anatomy reconstruction [26]. The limited evidence is probably related to technical difficulties and severe complications, including constriction and congestion of the vascular pedicle of the surgery, limiting its indications. Furthermore, the elasticity of the tissues in this region often does not justify a propeller flap.

#### 4.5.5. Free Flaps 

Free flaps represent an additional reconstructive strategy for FG, and various reconstructive alternatives could be considered. However, free flaps involve invasive surgery, require prolonged interventions, and incur considerable costs for the healthcare system. They are also technically difficult, require a highly specialized team, and should not represent first-line treatment. To the best of our knowledge, these are poorly adopted in FG. Only a case report of a fascia lata-free flap in pelvic exenteration for FG due to advanced rectal cancer is described in the present literature [89]. Despite the author’s valuable results, most FG patients often present with serious comorbidities, preventing invasive treatments [90,91]. Moreover, the inguinal-crural anatomical region offers versatile and effective reconstructions with less invasive strategies. Therefore, free flaps should be limited to selected cases, after excluding less invasive strategies. Their use should also be limited to highly specialized plastic surgery units. 

## 5. Future Prospects

Three-dimensional (3D) printing is emerging as a revolutionary technology for burn wounds and tissue reconstruction [92]. Specifically, it allows for a layer-by-layer deposition of cells and support materials, directly onto the injured areas, creating a 3D bioprinted graft [92]. This method could replace existing synthetic skin products, providing a more efficient and personalized solution for wound healing. Despite the limited experience, it could be extended to FG patients, especially in cases of extended loss of substance and limited skin availability.

## 6. Study Limitations

Study limitations include a limited focus on the recent literature. We only examined articles published between April 2014 and April 2024. However, this deliberate focus on recent studies was intentional, aiming to offer physicians a quick reference to the latest trends and developments in FG treatment, rather than a historical summary. Moreover, the included studies were considerably different from each other, preventing a true direct comparison. Precise data on epidemiological, clinical, and prognostic features were not extracted; moreover, no statistical analysis was performed. Nevertheless, the present review aimed to focus on FG reconstructive surgery options and recent trends, rather than assessing the feasibility and safety of each individual approach. Overall, we described the various therapeutic options available, based on the most recent scientific evidence and personal experience of the authors, aiming to improve its medical and surgical management.

## 7. Conclusions

FG remains a significant challenge in medical practice, primarily due to its diagnostic complexity, rapid progression, and considerable mortality rates. Comprehensive and immediate medical interventions, including broad-spectrum antibiotic therapy, surgical debridement, adjuvant therapies, and reconstructive surgery, appear to offer favorable results. The consequences of FG are particularly severe, up to penile degloving and testicular exposure. In the first instance, these are considered serious reconstructive challenges. However, the recent literature underscores a rising tendency towards the utilization of local random flaps or skin grafts as reconstructive treatments, when compared to more invasive locoregional flaps or free flaps. In this specific anatomical area, the former techniques are preferred for their versatility, feasibility, and reduced invasiveness. Indeed, the linguino-crural anatomical region has a good vascular supply and reliable surgical planes, allowing for minimally invasive surgical procedures, ultimately resulting in positive outcomes with limited complications.

Overall, although FG consequences seem catastrophic, they can be mostly managed with simple solutions, such as skin grafts and local random flaps. Several reconstructive options are available, but less invasive strategies should be preferred, starting with skin grafts and local random flaps. To date, no precise guidelines exist, but it is hoped that this study will represent a starting point for future research towards a recognized and approved protocol for the medical and surgical management of FG.

## Figures and Tables

**Table 1:** Clinical evidence for the appropriate surgical treatment for Fournier’s gangrene. N/A: not applicable.

Authors, Year	N. Patients	N. Males	N. Females	Mean Age, y	Defect	Comorbitidies	Surgical Treatment	Complications
Biju et al., 2023 [12]	34	27	7	58	12 Penile/Scrotal/Vulvar 22 Pernieal 0 Extra-perineal	18 Diabetes mellitus	1 Primary closure 5 Secondary intention 25 Skin grafts 3 Loco-regional flaps 0 Free flaps	N/A
Chen et al., 2023 [13]	36	31	5	53 (range 28–74)	N/A	18 Diabetes mellitus 10 Alcoholism 8 Smoking 4 Chronic renal failure 4 Cirrhosis 4 Obesity 3 Immunological diseases	0 Primary closure 30 Secondary intention 0 Skin grafts 6 Loco-regional flaps	N/A
Meuli et al., 2023 [14]	7	7	0	N/A	N/A	N/A	0 Primary closure 0 Secondary intention 0 Skin grafts 7 Loco-regional flaps (pedicled antero-lateral thigh flaps) 0 Free flaps	N/A
Maurya et al., 2022 [15]	6	N/A	N/A	N/A	N/A	N/A	0 Primary closure 0 Secondary intention 1 Skin grafts 5 Loco-regional flaps (scrotal flap, medial thigh flap, antero-lateral thigh flap, groin flap) 0 Free flaps	3 Wound infection 3 Wound dehiscence 2 Distal flap necrosis
Puranik et al., 2022 [16]	15	N/A	N/A	N/A	N/A	N/A	0 Primary closure 0 Secondary intention 1 Skin grafts 5 Loco-regional flaps (scrotal flap, super medial thigh flap, pudendal thigh flap, medial circumflex artery perforator flap, gracilis flap) 0 Free flaps.	N/A
Öcük et al., 2022 [17]	15	15	0	63 (range 32–87)	5 Penile/Scrotal 7 Pernieal 3 Extra-perineal	7 Diabetes mellitus 5 Cardiovascular diseases 2 Neoplasm 1 Immunological disease	0 Primary closure 0 Secondary intention 0 Skin grafts 15 Loco-regional flaps (superomedial fasciocutaneous thigh flap) 0 Free flaps	4 Wound dehiscence
Tripodi et al., 2022 [18]	23	23	0	44 (range 2–65)	N/A	N/A	0 Primary closure 0 Secondary intention 23 Skin grafts 0 Loco-regional flaps 0 Free flaps	N/A
Mortada et al., 2021 [19]	16	16	0	42 (range 20–61)	10 Penile/Scrotal 6 Pernieal 0 Extra-perineal	11 Diabetes mellitus 3 Cardiovascular diseases 2 Obesity	0 Primary closure 0 Secondary intention 16 Skin grafts 0 Loco-regional flaps 0 Free flaps	8 Wound dehiscence
Dadaci et al., 2021 [20]	29	29	0	64 (range 47–80)	29 Penile/Scrotal 0 Pernieal 0 Extra-perineal	15 Diabetes mellitus 2 Cardiovascular diseases 2 Chronic renal failure 1 Neoplasm	0 Primary closure 0 Secondary intention 0 Skin grafts 29 Loco-regional flaps (Limberg flap) 0 Free flaps	4 Seromas
Beecroft et al., 2021 [21]	31	25	6	N/A	N/A	N/A	0 Primary closure 0 Secondary intention NA Skin grafts NA Loco-regional flaps (gracilis flaps) 0 Free flaps	N/A
Agwu et al., 2020 [22]	35	N/A	N/A	N/A	N/A	N/A	16 Primary closure 10 Secondary intention 7 Skin grafts 2 Loco-regional flaps (advancement flaps) 0 Free flaps	N/A
Zhang et al., 2020 [23]	12	10	2	60 (range 45–66)	8 Penile/Scrotal/Vulvar 4 Pernieal 0 Extra-perineal	8 Diabetes mellitus 1 Neoplasm	1 Primary closure 4 Secondary intention 4 Skin grafts 3 Loco-regional flaps 0 Free flaps	2 Sepsis 2 Pneumonia 1 Renal failure 1 Heart failure
Khanal et al., 2020 [24]	14	14	0	41 (range 28–60)	N/A	3 Diabetes mellitus	0 Primary closure 0 Secondary intention 0 Skin grafts 16 Loco-regional flaps (bilateral pudendal flaps) 0 Free flaps.	1 Flap necrosis
Khanal, Bhawani et al., 2020 [24]	14	14	0	41 (range 28–60)	14 Scrotal 0 Pernieal 0 Extra-perineal	3 Diabetes mellitus	14 Loco-regional flaps	1 Flap necrosis
Garg et al., 2019 [25]	16	16	0	N/A	N/A	N/A	0 Primary closure 0 Secondary intention 16 Skin grafts 0 Loco-regional flaps 0 Free flaps	N/A
Louro et al., 2019 [26]	15	14	1	67 (range 46–86)	N/A	6 Diabetes mellitus 1 Chronic renal failure 1 Cirrhosis 1 Neoplasm 1 Hematological disease	0 Primary closure 0 Secondary intention 7 Skin graft 9 Loco-regional flaps (5 random flaps, 2 internal pudendal artery flaps, 1 McGregor propeller flap, 1 medial femoral circumflex artery perforator flap) 0 Free flaps	2 Flap dehiscence 2 Flap necrosis
Heijkoop et al., 2019 [27]	10	N/A	N/A	N/A	N/A	N/A	0 Primary closure 0 Secondary intention 9 Skin grafts 1 Loco-regional flap (rotational Flap 0 Free flaps	N/A
Ünverdi et al., 2019 [28]	13	13	0	54 (range 32–80)	13 Penile/Scrotal 0 Pernieal 0 Extra-perineal	N/A	0 Primary closure 0 Secondary intention 0 Skin grafts 13 Loco-regional flaps (internal pudendal artery perforator flap) 0 free flaps	1 Hematoma 1 Distal flap ecrosis
Lin et al., 2019 [29]	60	56	4	53	0 Penile/Scrotal/Vulvar 0 Pernieal 60 Extra-perineal	29 Diabetes mellitus 4 Chronic renal failure 2 Paraplegia	15 Primary closure 0 Secondary intention 45 Skin grafts 0 Loco-regional flap 0 Free flaps	1 Generalized sepsis 1 Wound infection 1 Massime hemorrhage 1 Septic death
Mello et al., 2018 [30]	15	15	0	48 (range 28–66)	N/A	N/A	0 Primary closure 0 Secondary intention 0 Skin grafts 15 Loco-regional flaps (superomedial fasciocutaneous thigh flap) 0 Free flaps	3 Wound dehiscence
El-Sabbagh et al., 2018 [31]	20	20	0	48 (range 37–59)	20 Penile/Scrotal 0 Pernieal 0 Extra-perineal	9 Diabetes mellitus 7 Bad hygiene 2 Chronic renal failure 1 Cirrhosis	4 Primary closure 0 Secondary intention 2 Skin grafts 8 Loco-regional flaps (pudendal thigh flap: 4 bilateral, 4 unilateral) 0 Free flaps	2 Wound infection 1 Seroma 1 Flap necrosis
Perry et al., 2018 [32]	17	11	6	49 (range 20–73)	2 Penile/Scrotal/Vulvar 5 Pernieal 10 Extra-perineal	12 Diabetes mellitus 9 Obesity 3 Cardiovascular diseases 3 Urologic disease	0 Primary closure 0 Secondary intention 2 Skin grafts 15 Loco-regional flaps (advancement flap) 0 Free flaps	N/A
Sockkalingam et al., 2018 [33]	17	N/A	N/A	N/A	N/A	N/A	13 Primary closure 0 Secondary intention 2 Skin grafts 2 Loco-regional flaps (prepucial skin flap) 0 Free flaps	N/A
Hahn et al., 2018 [34]	13	N/A	N/A	N/A	N/A	N/A	0 Primary closure 0 Secondary intention 8 Skin grafts 5 Loco-regional flaps (2 perforator flaps, 3 antero-lateral thigh flaps) 0 Free flaps	N/A
Hong et al., 2017 [7]	4	4	0	49 (range 40–63)	1 Penile/Scrotal 0 Perineal 3 Extra-perineal	N/A	0 Primary closure 0 Secondary intention 4 Skin grafts 0 Loco-regional flaps 0 Free flaps	1 Flap congestion 1 Wound dehiscence
Joon P. Hong et al., 2017 [35]	4	4	0	N/A	N/A	N/A	0 Primary closure 0 Secondary intention 0 Skin grafts 4 Loco-regional flaps (multiple pedicled perforator flaps) 0 Free flaps	1 Flap congestion 1 Wound dehiscence
Djedovic et al., 2017 [36]	8	8	0	57 (range 32–81)	3 Penile/Scrotal/Vulvar 5 Pernieal 0 Extra-perineal	5 Smoking 4 Diabetes mellitus 4 Cardiovascular diseases 3 Alcoholism	0 Primary closure 0 Secondary intention 0 Skin grafts 8 Loco-regional flaps (bilateral medial thigh lift) 0 Free flaps	2 Wound infection 1 Hematoma 1 Flap necrosis
Orhan et al., 2017 [37]	13	13	0	56 (range 46–72)	9 Penile/Scrotal 0 Perineal 4 Extra-perineal	22 Diabetes mellitus 7 Obesity 2 Chronic renal failure	0 Primary closure 0 Secondary intention 13 Skin grafts 0 Loco-regional flaps 0 Free flaps	N/A
Fatih Yanaral et al., 2017 [38]	20	20	0	N/A	N/A	N/A	0 Primary closure 0 Secondary intention NA Skin grafts NA Loco-regional flaps 0 Free flaps	N/A
Okwudili et al., 2016 [39]	12	12	0	38	12 Penile/Scrotal 0 Perineal 0 Extra-perineal	N/A	0 Primary closure 0 Secondary intention 0 Skin grafts 12 Loco-regional flaps (temporary subcutaneous thigh pouch) 0 Free flaps	N/A
Lin et al., 2016 [40]	10	10	0	59 (range 39–82)	1 Penile/Scrotal 8 Pernieal 1 Extra-perineal	8 Dibetes mellitus 4 Cirrhosis 2 Cardiovascular diseases 2 Alcoholism 2 Chronic renal failure 1 Paraplegia	0 Primary closure 0 Secondary intention 0 Skin grafts 10 Loco-regional flaps (antero-lateral thigh flaps) 0 Free flaps	1 Hematoma
Ludolph, Ingo et al., 2016 [41]	3	3	0	48 (range 39–60)	1 Penile/Scrotal 2 Perineal 0 Extra-perineal	1 Alcoholism 1 Smoking	3 Skin grafts	No complications
Konofaos et al., 2015 [42]	6	6	0	N/A	6 Penile/Scrotal 0 Perineal 0 Extra-perineal	N/A	0 Primary closure 0 Secondary intention 2 Skin grafts 0 Loco-regional flaps 0 Free flaps	No complications
Alwaal et al., 2015 [43]	13	N/A	N/A	N/A	N/A	N/A	0 Primary closure 0 Secondary intention 13 Skin grafts 0 Loco-regional flaps 0 Free flaps	N/A
Di Summa et al., 2015 [44]	4	4	0	50	N/A	8 Smoking 5 Alcoholism 2 Diabetes mellitus 1 Obesity 1 Chronic kidney failure	0 Primary closure 0 Secondary intention 0 Skin grafts 4 Loco-regional flaps (combined pedicled antero-lateral thigh and vastus lateralis flap 0 Free flaps	2 Wound dehiscence
Phillipo L. Chalya et al., 2015 [45]	84	82	2	43 (range 15–76)	66 Penile/Scrotal/Vulvar 4 Pernieal 14 Extra-perineal	14 Diabetes mellitus 9 Immulogical disease	0 Primary closure 64 Secondary intention 14 Skin grafts 5 Loco-regional flaps (rotation flap). 0 Free flaps.	N/A
Chao Li et al., 2015 [46]	20	20	0	N/A	N/A	N/A	0 Primary closure 0 Secondary intention 13 Skin grafts 7 Loco-regional flaps (scrotoplasty) 0 Free flaps	N/A
Oguz et al., 2015 [47]	43	34	9	52 (range 26–90)	N/A	18 Diabetes mellitus 3 Paraplegia 3 Cirrhosis 3 Chronic renal failure 1 Neoplasm	N/A Primary closure N/A Secondary intention N/A Skin grafts N/A Loco-regional flaps N/A Free flaps	N/A

**Table 2:** Fournier’s gangrene reconstruction.

Demographics	
N. patients	719
N. males	478 (92.6%)
N. females	37 (7.4%)
Average age	51 (range 15–90)
**Comorbidities**	Data available for 510/719 (71.4%)
Diabetes mellitus	207 (40.5%)
Cardiovascular disease	31 (6.0%)
Obesity	23 (4.5%)
Smoking	22 (4.3%)
Alcoholism	21 (4.1%)
Renal failure	19 (3.7%)
Immunosuppression	15 (2.5%)
Cirrhosis	13 (2.5%)
Paraplegia	6 (1.1%)
Neoplasm	6 (1.1%)
**Defect extension**	Data available for 370/719 (51.5%)
Penile/scrotal/vulvar	212 (57.8%)
Perineal	58 (15.7%)
Extra-perineal	100 (27.0%)
**Reconstructions**	Data available for 593/719 (82.5%)
Direct closure	35/593 (5.9%)
Healing by secondary intention	113/593 (19.1%)
Skin grafts	222/593 (37.4%)
Loco-regional flaps	223/593 (37.6%)
Random flaps	90/223 (40.4%)
Flaps based on known vascular anatomy	133/223 (59.6%)
Free flaps	0/593 (0%)
Minimally invasive reconstructive strategies (direct closure, secondary healing, grafts, and local random flaps)	460/593 (77.6%)
More invasive strategies (loco-regional flaps based on known vascular anatomy and free flaps)	133/593 cases (22.4%)
**Complications**	Data available for 272/719 (37.8%)
Wound dehiscence	27 (9.9%)
Flap necrosis	9 (3.3%)
Wound infection	8 (2.9%)
Seroma	5 (1.8%)
Hematoma	3 (1.1%)
Flap congestion Renal failure Pneumonia	2 (0.7%)
Generalised sepsis	4 (1.4%)
No complications	214 (78.7%)

## Data Availability

Data available on request due to privacy/ethical restrictions.

## References

[1] Fournier J.A. (1988). Jean-Alfred Fournier 1832–1914. Gangrene foudroyante de la verge (overwhelming gangrene). Sem Med 1883. Dis. Colon Rectum.

[2] Smith G.L., Bunker C.B., Dinneen M.D. (1998). Fournier’s gangrene. Br. J. Urol..

[3] Rodriguez Alonso A., Perez Garcia M.D., Nunez Lopez A., Ojea Calvo A., Alonso Rodrigo A., Rodriguez Iglesias B., Barros Rodriguez J.M., Benavente Delgado J., Nogueira March J.L. (2000). Fournier’s gangrene: Anatomo-clinical features in adults and children. Therapy update. Actas Urol. Esp..

[4] Eke N. (2000). Fournier’s gangrene: A review of 1726 cases. Br. J. Surg..

[5] Czymek R., Frank P., Limmer S., Schmidt A., Jungbluth T., Roblick U., Burk C., Bruch H.P., Kujath P. (2010). Fournier’s gangrene: Is the female gender a risk factor?. Langenbecks Arch. Surg..

[6] Yanar H., Taviloglu K., Ertekin C., Guloglu R., Zorba U., Cabioglu N., Baspinar I. (2006). Fournier’s gangrene: Risk factors and strategies for management. World J. Surg..

[7] Hong K.S., Yi H.J., Lee R.A., Kim K.H., Chung S.S. (2017). Prognostic factors and treatment outcomes for patients with Fournier’s gangrene: A retrospective study. Int. Wound J..

[8] Desai R., Batura D. (2023). A contemporaneous narrative review of Fournier’s gangrene. Urologia.

[9] Insua-Pereira I., Ferreira P.C., Teixeira S., Barreiro D., Silva A. (2020). Fournier’s gangrene: A review of reconstructive options. Cent. Eur. J. Urol..

[10] Huayllani M.T., Cheema A.S., McGuire M.J., Janis J.E. (2022). Practical Review of the Current Management of Fournier’s Gangrene. Plast. Reconstr. Surg. Glob. Open.

[11] Moher D., Liberati A., Tetzlaff J., Altman D.G., Group P. (2009). Preferred reporting items for systematic reviews and meta-analyses: The PRISMA statement. PLoS Med..

[12] Biju N.E., Sadiq M., Raj S., Patel A., Shah R., Weale R.D., Thomas K., Rose V. (2023). Fournier’s gangrene reconstruction: A 10-year retrospective analysis of practice at Guys and St Thomas’s NHS Foundation Trust. J. Plast. Reconstr. Aesthet. Surg..

[13] Chen J.H., Li Y.B., Li D.G., Zeng X.M., Yao Q.Y., Fu J., Wang G.H., Huang X.Y. (2023). Vacuum sealing drainage to treat Fournier’s gangrene. BMC Surg..

[14] Meuli J.N., Hubner M., Martineau J., Oranges C.M., Guillier D., Raffoul W., di Summa P.G. (2023). Impact of etiology leading to abdominoperineal resection with anterolateral thigh flap reconstruction: A retrospective cohort study. J. Surg. Oncol..

[15] Maurya R., Mir M.A., Mahajan S. (2022). Various Options for Scrotal Reconstruction: A Prospective Observational Study. Cureus.

[16] Puranik A., Baskaran S., Kumar R.R. (2022). An Innovative Technique of Testicular Preservation in Fournier’s Gangrene: Surgical Details and Illustration. Cureus.

[17] Ocuk O., Yagin F.H., Dinc O.G., Firat C. (2022). Effectiveness of Fasciocutaneous Superomedial Thigh Flap in Reconstruction of Fournier Gangrene Defects. Eplasty.

[18] Tripodi D., Guastafierro A., Gagliardi F., Amabile M.I., Lori E., Cirocchi R., Pironi D., Forte F., Cannistra C., Sorrenti S. (2022). A Retrospective Case Series in Fournier’s Disease: And Its Emergency Management et Grafting Technique for Penis Coverage. Emerg. Med. Int..

[19] Mortada H., Alhablany T., Alkahtani D., Rashidi M.E., Altamimi A. (2021). Meshed Versus Sheet Skin Graft for Scrotum and Perineal Skin Loss: A Retrospective Comparative Study. Cureus.

[20] Dadaci M., Yildirim M.E.C., Yarar S., Ince B. (2021). Assessment of Outcomes After Limberg Flap Reconstruction for Scrotal Defects in Patients With Fournier’s Gangrene. Wounds.

[21] Beecroft N.J., Jaeger C.D., Rose J.R., Becerra C.M.C., Shah N.C., Palettas M.S., Lehman A., Posid T., Jenkins L.C., Baradaran N. (2021). Fournier’s Gangrene in Females: Presentation and Management at a Tertiary Center. Urology.

[22] Agwu N.P., Muhammad A.S., Abdullahi A.A., Bashir B., Legbo J.N., Mungadi I.A. (2020). Pattern and outcome of management of Fournier’s gangrene in a resource-constraint setting. Urol. Ann..

[23] Zhang K.F., Shi C.X., Chen S.Y., Wei W. (2022). Progress in Multidisciplinary Treatment of Fournier’s Gangrene. Infect. Drug Resist..

[24] Khanal B., Agrawal S., Gurung R., Sah S., Gupta R. (2020). Pudendal flap-a good option for creating neo-scrotum after Fournier’s gangrene: A case series. J. Surg. Case Rep..

[25] Garg G., Singh V., Sinha R.J., Sharma A., Pandey S., Aggarwal A. (2019). Outcomes of patients with Fournier’s Gangrene: 12-year experience from a tertiary care referral center. Turk. J. Urol..

[26] Louro J.M., Albano M., Baltazar J., Vaz M., Diogo C., Ramos S., Cabral L. (2019). Fournier’s Gangrene: 10-Year Experience of a Plastic Surgery and Burns Department at a Tertiary Hospital. Acta Med. Port..

[27] Heijkoop B., Parker N., Spernat D. (2019). Fournier’s gangrene: Not as lethal as previously thought? A case series. ANZ J. Surg..

[28] Unverdi O.F., Kemaloglu C.A. (2019). A Reliable Technique in the Reconstruction of Large Penoscrotal Defect: Internal Pudendal Artery Perforator Flap. Urology.

[29] Lin H.-C., Chen Z.-Q., Chen H.-X., He Q.-L., Liu Z.-M., Zhou Z.-Y., Shi R., Ren D.-L. (2019). Outcomes in patients with Fournier’s gangrene originating from the anorectal region with a particular focus on those without perineal involvement. Gastroenterol. Rep..

[30] Mello D.F., Helene Junior A. (2018). Scrotal reconstruction with superomedial fasciocutaneous thigh flap. Rev. Col. Bras. Cir..

[31] El-Sabbagh A. (2018). Coverage of the scrotum after Fournier’s gangrene. GMS Interdiscip. Plast. Reconstr. Surg. DGPW.

[32] Perry T.L., Kranker L.M., Mobley E.E., Curry E.E., Johnson R.M. (2018). Outcomes in Fournier’s Gangrene Using Skin and Soft Tissue Sparing Flap Preservation Surgery for Wound Closure: An Alternative Approach to Wide Radical Debridement. Wounds.

[33] Sockkalingam V.S., Subburayan E., Velu E., Rajashekar S.T., Swamy A.M. (2018). Fournier’s gangrene: Prospective study of 34 patients in South Indian population and treatment strategies. Pan Afr. Med. J..

[34] Hahn H.M., Jeong K.S., Park D.H., Park M.C., Lee I.J. (2018). Analysis of prognostic factors affecting poor outcomes in 41 cases of Fournier gangrene. Ann. Surg. Treat. Res..

[35] Hong J.P., Kim C.G., Suh H.S., Kim H., Yoon C.S., Kim K.N. (2017). Perineal reconstruction with multiple perforator flaps based on anatomical divisions. Microsurgery.

[36] Djedovic G., Del Frari B., Matiasek J., Schiltz D., Engelhardt T.O., Pierer G., Rieger U.M. (2017). The versatility of the medial thigh lift for defect coverage in the genito-perineal region. Int. Wound J..

[37] Orhan E., Senen D. (2017). Using negative pressure therapy for improving skin graft taking on genital area defects following Fournier gangrene. Turk. J. Urol..

[38] Yanaral F., Balci C., Ozgor F., Simsek A., Onuk O., Aydin M., Nuhoglu B. (2017). Comparison of conventional dressings and vacuum-assisted closure in the wound therapy of Fournier’s gangrene. Arch. Ital. Urol. Androl..

[39] Okwudili O.A. (2016). Temporary Relocation of the Testes in Anteromedial Thigh Pouches Facilitates Delayed Primary Scrotal Wound Closure in Fournier Gangrene With Extensive Loss of Scrotal Skin-Experience With 12 Cases. Ann. Plast. Surg..

[40] Lin C.T., Chang S.C., Chen S.G., Tzeng Y.S. (2016). Reconstruction of perineoscrotal defects in Fournier’s gangrene with pedicle anterolateral thigh perforator flap. ANZ J. Surg..

[41] Ludolph I., Titel T., Beier J.P., Dragu A., Schmitz M., Wullich B., Horch R.E. (2016). Penile reconstruction with dermal template and vacuum therapy in severe skin and soft tissue defects caused by Fournier’s gangrene and hidradenitis suppurativa. Int. Wound J..

[42] Konofaos P., Hickerson W.L. (2015). A Technique for Improving Cosmesis After Primary Scrotum Reconstruction With Skin Grafts. Ann. Plast. Surg..

[43] Alwaal A., McAninch J.W., Harris C.R., Breyer B.N. (2015). Utilities of Split-Thickness Skin Grafting for Male Genital Reconstruction. Urology.

[44] di Summa P.G., Tremp M., Meyer Zu Schwabedissen M., Schaefer D.J., Kalbermatten D.F., Raffoul W. (2015). The Combined Pedicled Anterolateral Thigh and Vastus Lateralis Flap as Filler for Complex Perineal Defects. Ann. Plast. Surg..

[45] Chalya P.L., Igenge J.Z., Mabula J.B., Simbila S. (2015). Fournier’s gangrene at a tertiary health facility in northwestern Tanzania: A single centre experiences with 84 patients. BMC Res. Notes.

[46] Li C., Zhou X., Liu L.F., Qi F., Chen J.B., Zu X.B. (2015). Hyperbaric Oxygen Therapy as an Adjuvant Therapy for Comprehensive Treatment of Fournier’s Gangrene. Urol. Int..

[47] Oguz A., Gumus M., Turkoglu A., Bozdag Z., Ulger B.V., Agacayak E., Boyuk A. (2015). Fournier’s Gangrene: A Summary of 10 Years of Clinical Experience. Int. Surg..

[48] Sheehy S.A., Kelly M.E., Francis E.C., Sweeney K.J., Hussey A. (2016). A rare case of Fournier’s Gangrene. J. Surg. Case Rep..

[49] McDonagh T.A., Metra M., Adamo M., Gardner R.S., Baumbach A., Bohm M., Burri H., Butler J., Celutkiene J., Chioncel O. (2021). 2021 ESC Guidelines for the diagnosis and treatment of acute and chronic heart failure. Eur. Heart J..

[50] McDonagh T.A., Metra M., Adamo M., Gardner R.S., Baumbach A., Bohm M., Burri H., Butler J., Celutkiene J., Chioncel O. (2023). 2023 Focused Update of the 2021 ESC Guidelines for the diagnosis and treatment of acute and chronic heart failure. Eur. Heart J..

[51] Chowdhury T., Gousy N., Bellamkonda A., Dutta J., Zaman C.F., Zakia U.B., Tasha T., Dutta P., Deb Roy P., Gomez A.M. (2022). Fournier’s Gangrene: A Coexistence or Consanguinity of SGLT-2 Inhibitor Therapy. Cureus.

[52] Bersoff-Matcha S.J., Chamberlain C., Cao C., Kortepeter C., Chong W.H. (2019). Fournier Gangrene Associated With Sodium-Glucose Cotransporter-2 Inhibitors: A Review of Spontaneous Postmarketing Cases. Ann. Intern. Med..

[53] Wright E.M. (2021). SGLT2 Inhibitors: Physiology and Pharmacology. Kidney360.

[54] Brust-Sisti L., Rudawsky N., Gonzalez J., Brunetti L. (2022). The Role of Sodium-Glucose Cotransporter-2 Inhibition in Heart Failure with Preserved Ejection Fraction. Pharmacy.

[55] Tran B.A., Updike W.H., Bullers K., Serag-Bolos E. (2022). Sodium-Glucose Cotransporter 2 Inhibitor Use Associated With Fournier’s Gangrene: A Review of Case Reports and Spontaneous Post-Marketing Cases. Clin. Diabetes.

[56] Olivieri V., Ruggiero G., Abate D., Serra N., Fortunati V., Griffa D., Forte F., Corongiu E. (2020). Fatal infections in andrology. Atypical clinical presentation of a Fournier’s disease. Arch. Ital. Urol. Androl..

[57] Auerbach J., Bornstein K., Ramzy M., Cabrera J., Montrief T., Long B. (2020). Fournier Gangrene in the Emergency Department: Diagnostic Dilemmas, Treatments and Current Perspectives. Open Access Emerg. Med..

[58] Hasham S., Matteucci P., Stanley P.R., Hart N.B. (2005). Necrotising fasciitis. BMJ.

[59] Chennamsetty A., Khourdaji I., Burks F., Killinger K.A. (2015). Contemporary diagnosis and management of Fournier’s gangrene. Ther. Adv. Urol..

[60] Levenson R.B., Singh A.K., Novelline R.A. (2008). Fournier gangrene: Role of imaging. Radiographics.

[61] Azmi Y.A., Alkaff F.F., Renaldo J., Wirjopranoto S., Prasetiyanti R., Soetanto K.M., Salamah S., Purba A.K.R., Postma M.J. (2023). Comparison of different scoring systems for predicting in-hospital mortality for patients with Fournier gangrene. World J. Urol..

[62] Bowen D., Juliebo-Jones P., Somani B.K. (2022). Global outcomes and lessons learned in the management of Fournier’s gangrene from high-volume centres: Findings from a literature review over the last two decades. World J. Urol..

[63] Khan A., Gidda H., Murphy N., Alshanqeeti S., Singh I., Wasay A., Haseeb M. (2022). An Unusual Bacterial Etiology of Fournier’s Gangrene in an Immunocompetent Patient. Cureus.

[64] El-Shazly M., Aziz M., Aboutaleb H., Salem S., El-Sherif E., Selim M., Sultan M., Omar M., Abd Elbaky T., Zanaty F. (2016). Management of equivocal (early) Fournier’s gangrene. Ther. Adv. Urol..

[65] Matsuoka Y., Himejima T., Kakudo N. (2022). A New Treatment Strategy for Chronic Wounds Using an Ultrasonic Debridement Device: Indications and Limitations. Int. J. Surg. Wound Care.

[66] Tanuma T., Ishikawa S., Saito J., Kurihara T., Ichioka S. (2023). Ultrasonic Debridement of Fournier Gangrene. Plast. Reconstr. Surg. Glob. Open.

[67] Iacovelli V., Cipriani C., Sandri M., Filippone R., Ferracci A., Micali S., Rocco B., Puliatti S., Ferrarese P., Benedetto G. (2021). The role of vacuum-assisted closure (VAC) therapy in the management of FOURNIER’S gangrene: A retrospective multi-institutional cohort study. World J. Urol..

[68] Hung M.-C., Chou C.-L., Cheng L.-C., Ho C.-H., Niu K.-C., Chen H.-L., Tian Y.-F., Liu C.-L. (2015). The role of hyperbaric oxygen therapy in treating extensive Fournier’s gangrene. Urol. Sci..

[69] Feres O., Feitosa M.R., Ribeiro da Rocha J.J., Miranda J.M., Dos Santos L.E., Feres A.C., de Camargo H.P., Parra R.S. (2021). Hyperbaric oxygen therapy decreases mortality due to Fournier’s gangrene: A retrospective comparative study. Med. Gas Res..

[70] Raizandha M.A., Hidayatullah F., Kloping Y.P., Rahman I.A., Djatisoesanto W., Rizaldi F. (2022). The role of hyperbaric oxygen therapy in Fournier’s Gangrene: A systematic review and meta-analysis of observational studies. Int. Braz. J. Urol..

[71] Li Y., Qin C., Yan L., Tong C., Qiu J., Zhao Y., Xiao Y., Wang X. (2021). Urogenital fascia anatomy study in the inguinal region of 10 formalin-fixed cadavers: New understanding for laparoscopic inguinal hernia repair. BMC Surg..

[72] Tuma F., Lopez R.A., Varacallo M. (2024). Anatomy, Abdomen and Pelvis: Inguinal Region (Inguinal Canal). StatPearls.

[73] Raad G., Massaad V., Serdarogullari M., Bakos H.W., Issa R., Khachan M.J., Makhlouf N., Mourad Y., Fakih C., Fakih F. (2023). Functional histology of human scrotal wall layers and their overlooked relation with infertility: A narrative review. Int. J. Impot. Res..

[74] Tiwana M.S., Leslie S.W. (2024). Anatomy, Abdomen and Pelvis: Testes. StatPearls.

[75] Artas H., Orhan I. (2007). Scrotal calculi. J. Ultrasound Med..

[76] Kessler R. (1982). Vasectomy and vasovasostomy. Surg. Clin. N. Am..

[77] Karian L.S., Chung S.Y., Lee E.S. (2015). Reconstruction of Defects After Fournier Gangrene: A Systematic Review. Eplasty.

[78] Wong C.H., Wei F.C. (2010). Anterolateral thigh flap. Head Neck.

[79] Coskunfirat O.K., Uslu A., Cinpolat A., Bektas G. (2011). Superiority of medial circumflex femoral artery perforator flap in scrotal reconstruction. Ann. Plast. Surg..

[80] Hallock G.G. (2004). The conjoint medial circumflex femoral perforator and gracilis muscle free flap. Plast. Reconstr. Surg..

[81] Hallock G.G. (2006). Scrotal reconstruction following fournier gangrene using the medial circumflex femoral artery perforator flap. Ann. Plast. Surg..

[82] Morris S.F., Yang D. (1999). Gracilis muscle: Arterial and neural basis for subdivision. Ann. Plast. Surg..

[83] Monstrey S., Blondeel P., Van Landuyt K., Verpaele A., Tonnard P., Matton G. (2001). The versatility of the pudendal thigh fasciocutaneous flap used as an island flap. Plast. Reconstr. Surg..

[84] Bai J., Song J.X., Yang C. (2007). Pudendal-thigh flap: Anatomic basis and application in repairing and reconstructing male perineal region. Zhonghua Wai Ke Za Zhi.

[85] Rajput S., Kuruoglu D., Salinas C.A., Sen I., Kalra M., Moran S.L. (2023). Flap management of groin wounds following vascular procedures: A review of 270 flaps for vascular salvage. J. Plast. Reconstr. Aesthetic Surg..

[86] Ohtsuka H., Nakaoka H., Saeki N., Miki Y. (1985). Island groin flap. Ann. Plast. Surg..

[87] McGregor I.A., Jackson I.T. (1972). The groin flap. Br. J. Plast. Surg..

[88] Boissiere F., Luca-Pozner V., Vaysse C., Kerfant N., Herlin C., Chaput B. (2019). The SCIP propeller flap: Versatility for reconstruction of locoregional defect(✰). J. Plast. Reconstr. Aesthetic Surg..

[89] Sawayama H., Miyanari N., Sugihara H., Iwagami S., Mizumoto T., Kubota T., Haga Y., Baba H. (2017). A fascia lata free flap in pelvic exenteration for Fournier gangrene due to advanced rectal cancer: A case report. Surg. Case Rep..

[90] Schifano N., Castiglione F., Cakir O.O., Montorsi F., Garaffa G. (2022). Reconstructive surgery of the scrotum: A systematic review. Int. J. Impot. Res..

[91] Ahn D.K., Kim S.W., Park S.Y., Kim Y.H. (2014). Reconstructive strategy and classification of penoscrotal defects. Urology.

[92] Varkey M., Visscher D.O., van Zuijlen P.P.M., Atala A., Yoo J.J. (2019). Skin bioprinting: The future of burn wound reconstruction?. Burn. Trauma.

